# Oxytocin does not stand alone

**DOI:** 10.1098/rstb.2021.0047

**Published:** 2022-08-29

**Authors:** Philip T. Putnam, Steve W. C. Chang

**Affiliations:** ^1^ Department of Psychology, Yale University, New Haven, CT 06520, USA; ^2^ Department of Neuroscience, Yale University School of Medicine, New Haven, CT 06510, USA; ^3^ Kavli Institute for Neuroscience, Yale University School of Medicine, New Haven, CT 06510, USA; ^4^ Wu Tsai Institute, Yale University, New Haven, CT 06510, USA

## 1. Introduction

The neuropeptide oxytocin persists as a hot topic in neuroscience, with steadily increasing interest in both scientific literature [1] and popular culture during recent decades [2–5]. This attention is inspired not only by the fascinating workings of oxytocin itself, which span in breadth from underlying critical reproductive biology [6] to supporting high-level social behaviours [7–9], but also by the enormous potential of leveraging oxytocin as a therapeutic to enhance social cognition [10–13]. However, accompanying this excitement are well-justified critiques. These include the following: (i) our understanding of how oxytocin modulates social cognition is lacking in methodological rigour [14–17]; (ii) the effects of oxytocin treatment can be highly context-dependent [18,19]; (iii) we are still building a mechanistic understanding for how oxytocin impacts social behaviours at the neurobiological level [9,20–24]; (iv) developmental and life experience can drastically change the function of oxytocinergic systems [25–27]; and (v) we lack a single overarching theory to predict how oxytocin may modulate behaviour [28–31]. These critical examinations are fundamental for advancing our understanding of oxytocin, enabling the utilization of oxytocinergic mechanisms as a means to study the neurobiology of social behaviours [32,33] and as a curative tool to restore the deficits in social cognition observed across a variety of psychiatric disorders [34,35].

One critical perspective that has been lacking in the literature until recently is the examination of how oxytocin interacts with other neuromodulatory systems [36]. In the brain, no single region or neuromodulator is an island, entire of itself, and although the practical considerations of laboratory experiments present limits on what any single experiment can address, studying the manipulations of oxytocin in isolation may lead to an incomplete or incorrect understanding. Historical experiments [6,37–42] have laid the crucial and foundational bases for researchers today to move forward with the difficult, but necessary, tasks of examining the substrates and effects of oxytocin in increasingly naturalistic behavioural contexts [7,32,43–45] and from holistic perspectives [44]. Present incongruities in our understanding of oxytocin may be, in part, the result of experimental approaches that seek to isolate the oxytocin system as an experimental variable while ignoring the rest of the brain or body. This is not intended as a criticism of past experiments, which built our understanding of oxytocin from a uterine-contracting agent to our multi-faceted perspective today, but instead a proposition for future studies that seek to address as yet unanswered questions.

Indeed, the field is already successfully moving in the direction of examining oxytocin function under more naturalistic contexts and more holistically, aided by advancements in technology [46,47] and decades of critical introspection in the literature [14,15,17,48]. For example, recent research has identified supralinear enhancements of social gaze from combinatorial treatment of oxytocin and the opioid antagonist naloxone [49], demonstrating a mechanistic link between the oxytocinergic and opioidergic systems in the regulation of social attention. Moreover, in the mouse nucleus accumbens, the endogenous endocannabinoid anandamide binding at the CB1 receptors drives social reward, and blockade or selective activation of oxytocin neurons in the paraventricular nucleus of the hypothalamus can suppress or enhance this socially driven anandamide mobilization [50], connecting the endocannabinoid system with oxytocin. Likewise, the genetic deletion of presynaptic oxytocin receptors from the nucleus accumbens, removing the relevant serotonergic innervation to the nucleus accumbens, eliminates the rewarding aspects of social interaction in mice [51], suggesting a role for serotonin–oxytocin interactions in social behaviours. These and other mechanistic connections between oxytocin and other neuromodulators are beginning to demonstrate that many of the functions attributed to oxytocin are mediated through complex and sometimes powerful crosstalk between oxytocin and other neuromodulators. This exciting perspective will be crucial for future research examining not only oxytocin but also the neurobiology of social behaviour as a whole. Thus, the goal of this introduction, and this entire theme issue more broadly, is to highlight some of the known interactions between oxytocin and other neuromodulators and to provide a holistic perspective of oxytocin function for future studies.

## 2. Oxytocin and sex hormones

Oxytocin, and the closely related arginine-vasopressin, are the result of ancient gene duplication in vertebral evolution [52–55]. While contemporary neuroscience and psychology may emphasize the neuromodulatory role of oxytocin, the hormonal functions of oxytocin in reproductive behaviours [56], parturition [57], lactation [6] and early parent–infant interactions are undoubtedly the more primal functions for this complex non-apeptide. Given the role that oxytocin plays in reproductive biology, it is unsurprising that previous studies have linked oxytocin with other sex hormones in shaping behaviours. Intranasal administration of oxytocin, for example, will also increase blood plasma levels of testosterone in healthy men [58]. In males, intranasal oxytocin has been shown to blunt the correlation between testosterone reactivity and competitiveness [59]. Oxytocin administration can also modulate testosterone levels in fathers in a fashion that is correlated with father–child social behaviours such as social gaze and social touch [60].

In this theme issue, Bakermans-Kranenburg *et al*. [61] asks how hormonal levels fluctuate in men from pregnancy to after the birth of their firstborn child, and how oxytocin and other hormones could explain differences in the quality of their parenting. Both oxytocin and oestradiol remained stable from the pre- to post-natal periods, while vasopressin and testosterone declined. Interestingly, oxytocin by itself, or in relation to other hormones, was not related to paternal sensitivity. However, for fathers with high oestradiol, a higher level of testosterone was associated with lower sensitivity. Adding to this understanding, Jiang *et al*. [62] examined how both oxytocin and testosterone modulated the gaze of non-human primates when shown conspecific social and sexual images. Both oxytocin and testosterone increased the innate bias for gaze on female genitalia over female faces and promoted viewing of the forehead region where rhesus monkeys (*Macaca mulatta*) display sexual skin. This modulation of stimulus preference indicates that both oxytocin and testosterone influence reproductive behaviours by possibly increasing the visual salience of sexual features. Similarly, Paletta *et al*. [63] review how interactions between oxytocin and other regulatory systems mediate social behaviours, with a particular emphasis on the female sex hormone oestrogen, importantly highlighting a link between oxytocin and the oestrogen receptors in shaping behaviour.

## 3. Oxytocin interactions with dopaminergic, serotonergic and opioidergic systems

Interactions between oxytocin and other neuromodulator or neurotransmitter systems remain broadly underappreciated in the literature. However, links between oxytocin and the dopaminergic system are arguably the most well understood [64]. The classic prairie vole (*Microtus ochrogaster*) model of pair-bonding has been demonstrated to be mediated not just by oxytocin receptors in the nucleus accumbens [37–39,41], but also by mesolimbic dopamine circuits in the reward centres of the brain to create a conditioned partner preference [39,65]. Exploring this relationship between oxytocin binding and dopaminergic circuits, Frehner *et al*. [66] in this issue examined a dense clustering of oxytocin receptors in the human dopaminergic substantia nigra pars compacta to test if variations in oxytocin receptor expression could identify individuals with autism. Postmortem human brain tissue specimens revealed that females with autism had significantly lower levels of oxytocin receptor expression than did males with autism or typically developing males and females. *In situ* hybridization to visualize and quantify oxytocin receptor mRNA found no differences between groups, suggesting that the difference in receptor expression was possibly the result of local dysregulation in oxytocin receptor protein translation or changes in the endocytosis and recycling rates.

The nucleus accumbens is a key site of oxytocin–dopamine interactions, as detailed thoughtfully in a review by Borie and colleagues [67] that explores how endocannabinoids, by interacting with oxytocin, modulate experience-dependent social behaviours. Here, the authors examine how oxytocin modulates glutamatergic signalling through the recruitment of endocannabinoids in the prairie vole nucleus accumbens and broadly review our understanding of the effects of oxytocin–endocannabinoid interactions on social behaviour, with an emphasis on how sex differences and life experiences may modulate these processes.

Examining the relationship that oxytocin has with the dopamine and serotonin systems in maternal behaviours, Grieb & Lonstein [68] in this issue shed light on how these processes regulate different aspects of caregiving and postpartum behaviours. Although oxytocin–dopamine interactions have been understood to motivate active caregiving behaviours, such as retrieval of pups, the authors highlight the underappreciated interactions between oxytocin and serotonin. These oxytocin–serotonin interactions, they argue, regulate many of the remaining dimensions of maternal care including nursing, anxiety-like behaviours and strategies for coping with stress.

Beyond the classical neurotransmitters, oxytocin also interacts with other neuromodulatory systems. One notable example is interactions between oxytocin and the opioid receptor system, observed both *in vitro* [69–73] and in social behavioural experiments [49]. Putnam & Chang [74] detail interactions between the oxytocinergic and opioidergic systems in shaping social behaviours, postulating a model to explain the supralinear effects arising from co-administering oxytocin and opioid blockade on social attention.

## 4. Interventional oxytocin

The promise of using oxytocin as a therapeutic intervention remains a tantalizing goal for the field of social neuroscience [12,75]. However, a robustly effective approach has not yet been identified [48,76,77]. The paper by Wei *et al*. [78] in this issue tackles this problem by suggesting a combinatorial approach to treat neuropsychiatric social impairment using both the oxytocin and endocannabinoid systems. Here, the authors detail the neurobiology of both the oxytocin and the endocannabinoid systems and explain how a multi-targeted treatment strategy may be best suited to modulate the multiple signalling processes underlying social cognition.

In an intervention-focused research article, Daughters *et al*. [79] compare the efficacy of oxytocin treatment against a validated emotion training program to test improvements in emotion recognition. Interestingly, they show that psychological intervention, but not intranasal oxytocin, was able to improve recognition specifically for angry expressions. This interesting finding highlights that behavioural or psychological treatments, with fewer caveats and risks, could sometimes exceed pharmacological interventions and should be considered carefully for future clinical trials involving oxytocin.

## 5. Oxytocin interactions beyond the neuron

A holistic understanding of oxytocin extends beyond specific neurotransmitters or brain regions but encompasses underappreciated aspects of neurobiology. A prime example of this is reviewed by Gonzalez and Hammock [80], who examine how oxytocin interacts with the microglia. Here, the authors detail how microglia have a bidirectional regulatory relationship with the oxytocin system and how these processes enhance experience-dependent circuits during sensitive periods to shape social behaviours. This perspective not only emphasizes the important and undervalued role that microglia play in shaping neural activity [81], but also how microglia–oxytocin interactions may critically impact our understanding of endogenous oxytocin function [82].

Similarly, a review piece by Carter & Kingsbury [83] offers a novel perspective with respect to how the oxytocin system played an important evolutionary role in managing oxidative stress and inflammation from the earth's oxygen-rich conditions. The resultant unique properties of the oxytocin system may have significantly enabled vertebrates to manage the consequences of oxygen-linked processes, such as inflammation or free radicals, while still supporting complex social behaviours.

At the neurobiological level, ageing also drastically impacts cognitive capacities. However, the link with neuropeptides is understudied. Polk and colleagues [84] examine the link between oxytocin and social cognition in ageing, finding that higher levels of plasma oxytocin were associated with lower accuracy in emotion identification, while plasma levels of arginine-vasopressin had no relation to emotion identification accuracy. These novel findings support the involvement of oxytocin in age-related neural processes and the possible interactions between oxytocin and basic cognitive capacities.

Oxytocin interactions also encompass broad life events, as expounded by Bales and Rodgers [85] in a review examining oxytocin interactions in partner loss. The authors survey what is known about the neuroendocrine mechanisms that regulate the emotional consequences of partner loss and specifically focus on interactions among oxytocin, corticotropin-releasing-hormone and the κ-opioid system.

## 6. Conclusion

The final paper of our theme issue by Leng *et al*. [86] takes a critical perspective on whether oxytocin is indeed a ‘social’ neuropeptide. This is a fitting final note to this Introduction since our perspective on oxytocin must constantly be reevaluated and challenged. Without disputing the significance of previous findings, we suggest that future studies should seek to examine the function of oxytocin not in isolation, but instead from a holistic perspective. The interactions between oxytocin and other neuromodulatory systems in the brain and body are not only crucial to understanding oxytocinergic function, but also the fundamental neural substrates of social behaviour. Understanding these connections will enable scientists and clinicians to better realize therapeutic interventions targeting the oxytocinergic system and will provide a window into the evolved process that shaped social functions in a wide array of animal species.

## Data Availability

This article has no additional data.
